# Pharmacokinetics and pharmacodynamics of ticagrelor in subjects on hemodialysis and subjects with normal renal function

**DOI:** 10.1007/s00228-018-2484-7

**Published:** 2018-05-30

**Authors:** Renli Teng, Sharen Muldowney, Yonggang Zhao, Jolene Kay Berg, Jonathan Lu, Naeem D. Khan

**Affiliations:** 1 AstraZeneca, Gaithersburg, MD USA; 2 Careceutics LLC, 2016 Saint Andrews Dr, Berwyn, PA 19312 USA; 3 AstraZeneca, Wilmington, DE USA; 4 Skyview Research, Philadelphia, PA USA; 5 DaVita Clinical Research, Minneapolis, MN USA

**Keywords:** Hemodialysis, Pharmacokinetics, Pharmacodynamics, Ticagrelor

## Abstract

**Purpose:**

This single-dose, randomized, open-label, parallel-group, and crossover study assessed pharmacokinetics (PK), pharmacodynamics (PD), and safety of ticagrelor in subjects on hemodialysis versus healthy subjects.

**Methods:**

Hemodialysis subjects were randomized, receiving a single ticagrelor 90-mg dose 1 day post-hemodialysis or just before hemodialysis, with an intervening washout of ≥ 7 days. Healthy subjects (creatinine clearance ≥ 90 mL/min) received a single ticagrelor 90-mg dose. PK, PD (P2Y_12_ reaction units [PRU], inhibition of platelet aggregation [IPA]), and safety were evaluated.

**Results:**

Twenty-seven subjects (14 hemodialysis, 13 healthy) received ticagrelor. The mean maximum plasma concentration (*C*_max_) and area under the plasma concentration curve from time zero to infinity (AUC_0-∞_) of ticagrelor were 598.4 ng/mL and 3256.1 ng·h/mL, respectively, in pre-hemodialysis subjects; 560.3 ng/mL and 3015.1 ng·h/mL, respectively, in post-hemodialysis subjects; and 370.8 ng/mL and 2188.8 ng·h/mL, respectively, in healthy subjects. *C*_max_ and AUC_0-∞_ of AR-C124910XX, the active metabolite, were 152.3 ng/mL and 1144.2 ng·h/mL, respectively, in pre-hemodialysis subjects; 130.8 ng/mL and 1127.8 ng·h/mL, respectively, in post-hemodialysis subjects; and 111.7 ng/mL and 1000.4 ng·h/mL, respectively, in healthy subjects. Mean IPA time curves over 24 h post-dose were almost indistinguishable for all three treatments. The greatest reduction in mean PRU occurred approximately 2 h post-dose for all three treatments. No safety or tolerability issues were identified.

**Conclusion:**

Hemodialysis resulted in modestly higher exposure to ticagrelor and AR-C124910XX, with no clinically significant effect on PD or tolerability. Accordingly, no dose adjustment is required for hemodialysis patients. Timing of hemodialysis has little impact on ticagrelor PK, or the effect of ticagrelor on IPA.

**Electronic supplementary material:**

The online version of this article (10.1007/s00228-018-2484-7) contains supplementary material, which is available to authorized users.

## Introduction

Ticagrelor is a direct-acting, reversibly binding oral P2Y_12_ receptor antagonist that inhibits adenosine diphosphate (ADP)-mediated platelet aggregation [1]. It is given in combination with low-dose aspirin for the secondary prevention of atherothrombotic events in patients with acute coronary syndromes (ACS) [2–4].

Ticagrelor is primarily eliminated via hepatic metabolism, with renal excretion playing only a minor role. The primary route of excretion for the active metabolite of ticagrelor is most probably via biliary secretion [5, 6]. The pharmacokinetics (PK), pharmacodynamics (PD), and safety of ticagrelor have been previously studied in a variety of special populations, including subjects with severe renal impairment not on dialysis [7] and subjects with ACS and chronic kidney disease [8]. Compared with subjects with normal renal function, the maximum observed plasma concentration (*C*_max_) and area under the plasma concentration curve from time zero to infinity (AUC_0-∞)_ of ticagrelor were 20% lower in subjects with severe renal impairment, a difference that is not considered clinically significant. Likewise, PD measures of platelet aggregation appeared to be generally comparable in healthy subjects and those with severe renal impairment, and the safety profile was similar, with no dose adjustments required for renally impaired subjects [7]. When assessed pharmacodynamically versus clopidogrel in patients with ACS and chronic kidney disease, ticagrelor was again shown to be effective and was associated with greater reductions in P2Y_12_ platelet reaction units (PRU) in the 24 h following loading dose [8].

The PK, PD, and safety of ticagrelor are yet to be definitively established in subjects with end-stage renal disease (ESRD) receiving renal replacement therapy with hemodialysis. Consequently, there are no dosing recommendations for ticagrelor in these patients, despite their increased risk for atherothrombotic and bleeding events when compared with the general population [9–11].

This study was conducted to determine the effects of hemodialysis on the PK, PD (based on inhibition of platelet aggregation [IPA] and platelet reactivity, reported as PRU), safety, and tolerability of ticagrelor in subjects with ESRD on hemodialysis, and to provide a rational quantitative basis for ticagrelor dosing recommendations in this patient population.

## Methods

### Study design and treatment

This was a single-dose, randomized, open-label, parallel-group, and crossover study (NCT02022748) of healthy adult subjects with normal renal function and subjects on hemodialysis enrolled at two study centers (in Lakewood, Colorado and Minneapolis, Minnesota) in the USA. The study was conducted in accordance with the ethical principles that have their origin in the Declaration of Helsinki and in compliance with the International Conference on Harmonisation/Good Clinical Practice guidelines, AstraZeneca bioethics policy, and other applicable regulatory requirements. The study protocol was approved by an institutional review board for each study center, and written informed consent was obtained from all subjects.

Subjects with normal renal function (creatinine clearance ≥ 90 mL/min) were matched by age, weight, and sex to subjects with ESRD on hemodialysis. The main inclusion criteria were men or women aged 18–80 years, body weight ≥ 50 kg, and body mass index (BMI) 18–40 kg/m^2^, with normal renal function or suffering from ESRD requiring maintenance hemodialysis. Major exclusion criteria included pregnancy; lactation; indication for oral anticoagulant or antiplatelet therapy during the study period (low-dose aspirin was allowed for hemodialysis subjects only); history of ACS within 12 months of study start; contraindication to ticagrelor; increased bleeding risk (platelet count < 100,000/μL) or hemoglobin < 9 g/dL; concomitant therapy with strong cytochrome P450 3A (CYP3A) inhibitors, inducers, or substrates with a narrow therapeutic index within 14 days of study initiation; history of alcohol, substance, or drug abuse within the year preceding the study; and clinically significant laboratory abnormalities as judged by the investigator.

Subjects were screened within 21 days of study initiation (visit 1), which included a physical examination, clinical laboratory testing, 12-lead electrocardiogram (ECG), and relevant medical and surgical history. Renal function was estimated using the Cockcroft-Gault formula [12] and was used to confirm group placement at screening. Laboratory assessments were repeated prior to receiving the study drug. Subjects with normal renal function received a single oral ticagrelor 90-mg dose. Hemodialysis subjects received a single oral ticagrelor 90-mg dose in randomized order either 1 day following hemodialysis (post-hemodialysis) or just prior to the start of hemodialysis (pre-hemodialysis), with crossover to the other regimen after a washout period of at least 7 days.

All subjects were required to fast (2 h for hemodialysis subjects, 8 h overnight for healthy patients) prior to ticagrelor administration and for 2 h post-dose. Ticagrelor was administered with 120 and 240 mL of non-refrigerated water in hemodialysis and healthy subjects, respectively. Subjects sat in an upright or semi-recumbent position for at least 2 h following dosing. Water consumption was restricted from 2 h prior to 2 h following ticagrelor dosing.

### Sample collection

Venous blood samples were collected at 1 (for PK only), 2, 4, 6, 12, 24, 36, and 48 h post-dose. PK samples were collected into lithium heparin tubes, chilled, and centrifuged (10 min at 4 °C, relative force of 1500 g) within 30 min of sample collection. PD samples were collected in Greiner Bio-One Vacuette tubes (Greiner Bio-One North America Inc., Monroe, NC, USA), allowed to set for a minimum of 10 min, and assayed within 4 h of collection.

### PK and PD sample analyses

Samples for determination of ticagrelor and AR-C124910XX (active metabolite) concentrations in plasma were analyzed by Covance Inc. on behalf of AstraZeneca Research and Development, using an appropriate bioanalytical method [13].

### PK analyses

The PK parameters were estimated using standard non-compartmental methods and determined in the subjects who received a dose of ticagrelor, had PK data available, and had no major protocol deviations that might affect the PK of ticagrelor or AR-C124910XX. The following PK parameters were estimated for ticagrelor and AR-C124910XX: *C*_max_, AUC_0-∞_, time to reach maximum plasma concentration (*t*_max_), terminal elimination half-life (*t*_1/2_), and metabolite/parent drug *C*_max_ and AUC ratios. PK parameters were estimated using WinNonlin version 6.3 (Pharsight Corporation, Mountain View, CA, USA).

### PD analyses

The PD profile of ticagrelor was determined in the subjects who received a dose of ticagrelor, had PD data available, and had no major protocol deviations that might affect evaluation of PD, and was based on IPA and platelet reactivity. IPA was measured by light transmission aggregometry using 20-μM ADP as the agonist [14]. The peak IPA (IPA_max_) was estimated as the highest IPA (final extent); time to IPA_max_ was also assessed. The area under the effect curves from 0 to 48 h of final extent IPA (AUEC_0–48, IPA_) were calculated from IPA time curves using the linear trapezoid rule. IPA percentage was calculated at each time point using the following formula: 100 × (PA_BL_ − PA_*T*_)/PA_BL_, where PA_*T*_ is the mean platelet aggregation response at time *T*, and PA_BL_ is the mean response at pre-dose on day 1. The relationship between ticagrelor plasma concentrations and IPA was investigated using a sigmoid maximum effect (*E*_max_) model: IPA = *E*_max_*C*^*γ*^/(*C*^*γ*^ + EC_50_^*γ*^), where *E*_max_ is the maximum effect, EC_50_ is the concentration that produces 50% of maximal effect, gamma (*γ*) is the sigmoidicity or shape factor, and *C* is the plasma concentration of ticagrelor.

Platelet reactivity was measured with the VerifyNow™ P2Y_12_ assay (Accriva Diagnostics, San Diego, CA, USA), with results reported in PRU. The minimum PRU value (PRU_min_) was estimated as the lowest PRU value. The area under the effect curves from 0 to 48 h of final PRU (AUEC_0–48, PRU_) were calculated from PRU time curves using the linear trapezoid rule.

### Safety and tolerability

Assessments of safety and tolerability were based on the safety population (comprising all patients who received a dose of ticagrelor) and included monitoring of adverse events (AEs), physical examination, 12-lead ECGs, vital signs, and laboratory testing. AEs were monitored from time of informed consent until follow-up.

### Statistical analyses

PK parameters were summarized using descriptive statistics. All other analyses were generated using SAS® version 9.3 (SAS Institute, Inc., Cary, NC, USA). Treatment comparisons between healthy and hemodialysis subjects were performed using general linear methods with treatment effects. Comparisons of the two hemodialysis regimens were performed using a mixed model. Descriptive analyses were performed for each PD parameter, but the study was not powered for statistical analysis of PD. Sample size determinations were based on accepted standards for this type of investigation.

## Results

### Demographics and disposition

Twenty-seven subjects (14 hemodialysis subjects, 13 healthy subjects) received the study drug and underwent study procedures between October 2015 and May 2016. Three hemodialysis subjects discontinued treatment (two who received the pre-hemodialysis regimen first and one who received the post-hemodialysis regimen first). In two subjects, discontinuations were due to AEs, while the third subject chose to discontinue the study after receiving the first ticagrelor dose. All 13 healthy subjects completed treatment.

Healthy and hemodialysis subjects were generally well matched. The hemodialysis group comprised 12 men and 2 women, and had a mean (standard deviation [SD]) age of 50.6 (12.5) years and a mean (SD) BMI of 27.8 (4.2) kg/m^2^. The healthy subject group comprised 10 men and 3 women, with a mean (SD) age and BMI of 43.8 (10.4) years and 28.3 (3.8) kg/m^2^, respectively. In the hemodialysis group, five subjects were white and nine were black. Of the healthy subjects, six were white, five were black, and two were of other race.

All hemodialysis subjects were on concomitant medication, including treatments for hyperparathyroidism (doxercalciferol) and anemia (saccharated iron oxide, erythropoiesis-stimulating agents), and anticoagulants (heparin). One hemodialysis subject had taken amlodipine within 14 days of study initiation, and although this was recorded as a protocol deviation, it was considered to have negligible impact on the PK of ticagrelor and the subject was included in the PK analysis population. Two healthy subjects reported taking concomitant medications, which included acetaminophen, antacids (calcium carbonate), and aspirin.

### Pharmacokinetics

**Fig. 1:**
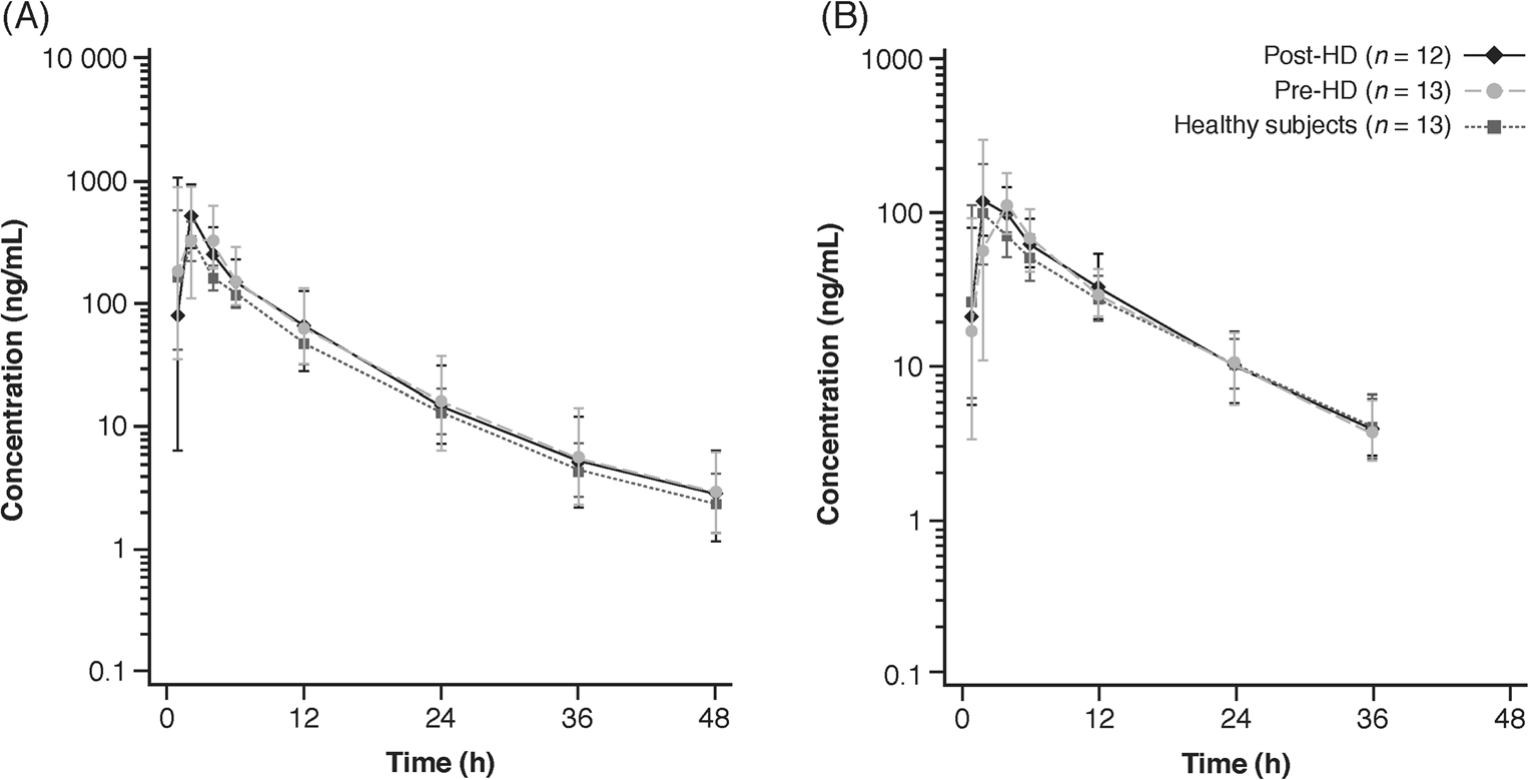
Mean (SD) of plasma concentration time curves for **a** ticagrelor and **b** AR-C124910XX for hemodialysis and healthy subjects. HD hemodialysis, SD standard deviation

The mean concentration–time profiles of ticagrelor and AR-C124910XX were comparable in hemodialysis and healthy subjects (Fig. 1), and the timing of ticagrelor dosing relative to hemodialysis did not appear to impact the plasma concentration profile.

Ticagrelor was rapidly absorbed, with a median *t*_max_ of 2 h in both hemodialysis and healthy subjects, and the mean *t*_1/2_ was comparable for all treatments (Table 1). The mean *C*_max_ of ticagrelor was 61% higher in subjects receiving the pre-hemodialysis ticagrelor treatment, compared with that in healthy subjects, and 51% higher following the post-hemodialysis regimen than that in healthy subjects (Table 1, Table S1). Likewise, the mean AUC_0-∞_ of ticagrelor was 49 and 38% higher when ticagrelor was given pre- and post-hemodialysis, respectively, than that in healthy subjects (Table 1, Table S1). The mean *C*_max_ and AUC_0-∞_ of ticagrelor were comparable for the two hemodialysis treatments (Table 1). The variability (CV%) for both *C*_max_ and AUC_0-∞_ of ticagrelor was approximately 50% in hemodialysis subjects, compared with 37% for *C*_max_ and 23% for AUC_0-∞_ in healthy subjects.

**Table 1:** Pharmacokinetic (PK) parameters for ticagrelor and ARC-124910XX in hemodialysis and healthy subjects

PK parameter^a^	Pre-HD (*n* = 13)	Post-HD (*n* = 12)	Healthy subjects (*n* = 13)
Ticagrelor			
*C*_max_, ng/mL	598.4 (47.9)	560.3 (54.0)	370.8 (37.3)
AUC_0-∞_, ng·h/mL	3256.1 (52.5)	3015.1 (54.2)	2188.8 (22.6)
*t*_max_, h	2.0 (1.0–4.0)	2.0 (1.0–4.0)	2.0 (1.0–6.0)
*t*_1/2_, h	8.4 (24.8)	8.2 (16.2)	8.3 (14.9)
AR-C124910XX			
*C*_max_, ng/mL	152.3 (54.3)	130.8 (38.3)	111.7 (60.0)
AUC_0-∞_, ng·h/mL	1144.2 (36.2)	1127.8 (39.3)	1000.4 (33.2)
*t*_max_, h	2.0 (2.0–4.0)	2.0 (2.0–4.0)	2.0 (2.0–6.0)
*t*_1/2_, h	7.4 (24.8)	7.3 (28.2)	8.4 (22.3)

*AUC*_*0-∞*_, area under the concentration curve (AUC) from time zero to infinity; *C*_*max*_, maximum observed plasma concentration; *HD*, hemodialysis; *t*_*1/2*_, terminal elimination half-life; *t*_*max*_, time to reach maximum plasma concentration

^a^Values are geometric mean (percentage coefficient of variation) for *C*_max_, AUC_0-∞_; median (range) for *t*_max_; arithmetic mean (standard deviation) for t_1/2_

AR-C124910XX was rapidly formed, with a median *t*_max_ of 2 h in both hemodialysis and healthy subjects, and the mean *t*_1/2_ was similar for all treatments (Table 1). In addition, the mean *C*_max_ of AR-C124910XX was 36% higher following the pre-hemodialysis regimen, compared with that in healthy subjects (Table 1, Table S1). The mean *C*_max_ of AR-C124910XX in patients receiving post-hemodialysis treatment was 17% higher versus that in healthy subjects. The mean AUC_0-∞_ of AR-C124910XX was 14 and 13% higher in subjects receiving ticagrelor pre- and post-hemodialysis, respectively, than that in healthy subjects (Table 1, Table S1), and like ticagrelor itself, exposure to AR-C124910XX was similar for the two hemodialysis treatment schedules. *C*_max_ of AR-C124910XX values displayed minor variability between the two hemodialysis regimens (pre-hemodialysis, 54%; post-hemodialysis, 38%). The variability of AUC_0-∞_ of AR-C124910XX was approximately 40% for both hemodialysis treatment schedules. The variability of *C*_max_ and AUC_0-∞_ of AR-C124910XX was 60 and 33%, respectively, for healthy subjects. The variability of *C*_max_ and AUC_0-∞_ of AR-C124910XX was considered to be generally similar for hemodialysis versus healthy subjects.

The mean metabolite-to-parent ratios for *C*_max_ (range, 0.26–0.33) and AUC_0-∞_ (range, 0.38–0.50) were also generally similar between hemodialysis and healthy subjects, suggesting that the presence of ESRD requiring hemodialysis and the timing of hemodialysis have little influence on the metabolic conversion of ticagrelor to AR-C124910XX.

### Pharmacodynamics

**Fig. 2:**
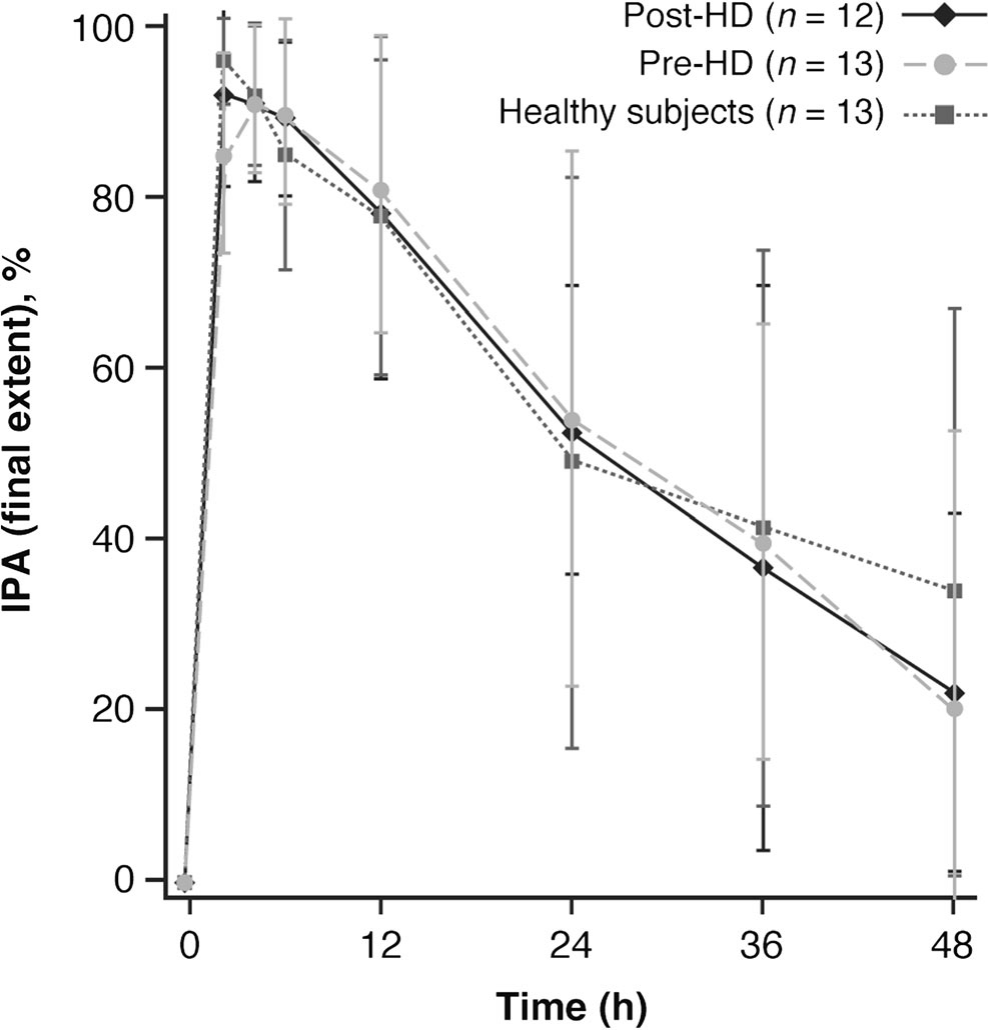
Mean (SD) inhibition of platelet aggregation (IPA) time curves for ticagrelor in hemodialysis and healthy subjects. HD hemodialysis, SD standard deviation

**Fig. 3:**
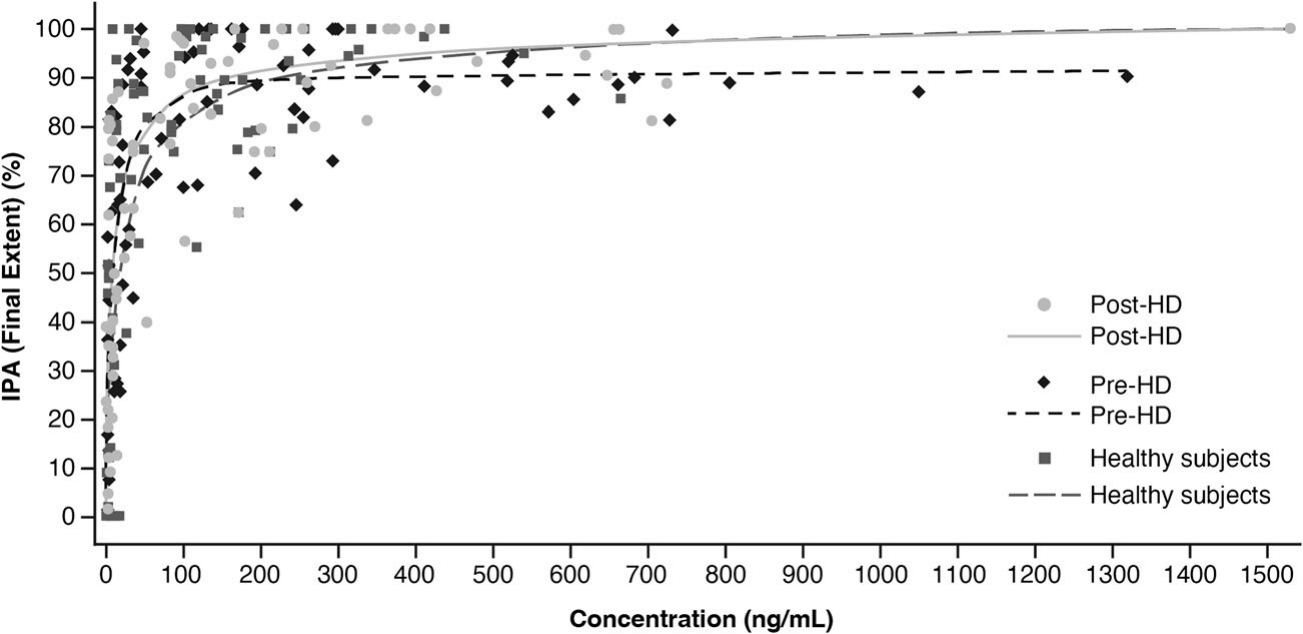
Inhibition of platelet aggregation (IPA) concentration curves for ticagrelor in hemodialysis and healthy subjects. HD hemodialysis

The mean IPA time curves over 24 h post-dose were almost indistinguishable for all three treatments (Fig. 2). Moreover, final extent IPA was over 90% for all three treatments approximately 2 h after ticagrelor administration (Fig. 2). The mean IPA_max_ and the AUEC_0–48, IPA_ were generally similar for all three treatments (Table 2), indicating that the presence of ESRD on hemodialysis and the timing of hemodialysis have little influence on the effect of ticagrelor on IPA. The median time to IPA_max_ was longer when ticagrelor was administered just prior to hemodialysis (4 h) than 1 day after hemodialysis (2 h) or in healthy subjects (2 h) (Table 2). Time to IPA_max_ displayed much greater variability in hemodialysis subjects (range 2–12 h for both treatments) than in healthy subjects (2 h in all 13 subjects) (Table 2). The *E*_max_ model parameters describing the PK/PD relationship between plasma concentrations of ticagrelor and IPA were comparable across all three treatments (Fig. 3), suggesting that the relationship between ticagrelor concentrations and IPA is not altered in ESRD patients on hemodialysis (Table 2).

**Table 2:** Pharmacodynamic (PD) parameters for ticagrelor in hemodialysis and healthy subjects

PD parameter^a^	Pre-HD (*n* = 13)	Post-HD (*n* = 12)	Healthy subjects (*n* = 13)
IPA_max_, %	92.8 (9.3)	95.5 (7.6)	95.9 (5.3)
TIPA_max_, h	4.0 (2.0–12.0)	2.0 (2.0–12.0)	2.0 (2.0–2.0)
AUEC_0–48, IPA_, %·h	2576.4 (32.1)	2550.3 (27.3)	2472.4 (46.0)
*E*_max_, %	92.1 (4.3)	105.3 (10.4)	101.3 (10.4)
EC_50_, ng/mL	9.0 (1.4)	15.5 (6.4)	8.4 (3.4)
Gamma	1.1 (0.2)	0.7 (0.1)	0.7 (0.2)
PRU_min_, PRU	83.8 (115.3)	33.4 (265.3)	7.3 (170.9)
TPRU_min_, h	2.0 (2.0–12.0)	2.0 (2.0–4.0)	2.0 (2.0–6.0)
AUEC_0–48, PRU_, PRU·h	12,440.0 (26.2)	12,577.2 (30.5)	7144.9 (42.9)

*AUEC*_*0–48, IPA*,_ area under the effect curve 0 to 48 h inhibition of platelet aggregation; *AUEC*_*0–48, PRU*,_ area under the effect curve 0 to 48 h P2Y_12_ reaction units; *EC*_*50*_, concentration that produces 50% maximum effect; *E*_*max*_, maximum effect; *Gamma*, sigmoidicity or shape factor; *HD*, hemodialysis; *IPA*_*max*_, maximum inhibition of platelet aggregation; *PRU*_*min*_, minimum P2Y_12_ reaction units; *TIPA*_*max*_, time to IPA_max_; *TPRU*_*min*_, time to PRU_min_

^a^Values are geometric mean (percentage coefficient of variation) for IPA_max_, AUEC_0–48, IPA_, PRU_min_, and AUEC_0–48, PRU_; median (range) for TIPA_max_ and TPRU_min_; estimate (standard error) for *E*_max_, EC_50_, and gamma

**Fig. 4:**
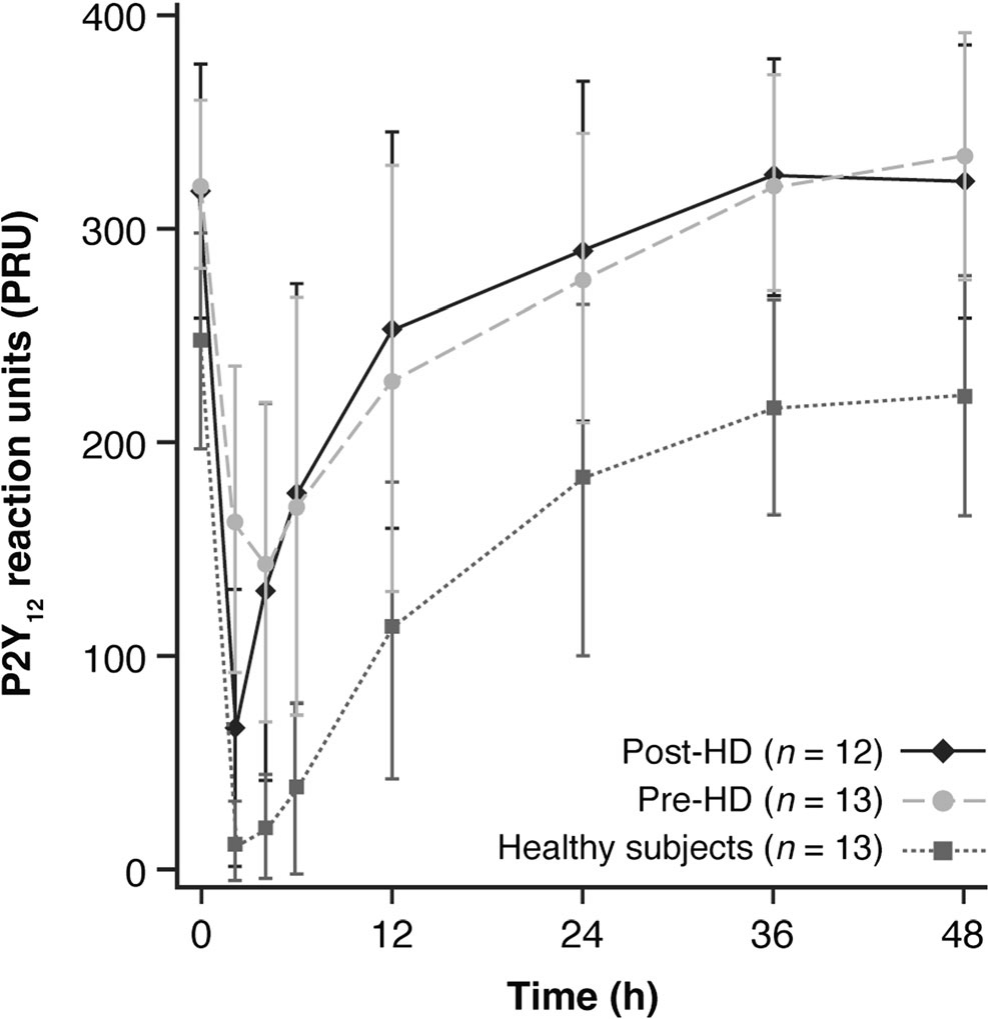
Mean (SD) P2Y_12_ reaction unit (PRU) time curves for ticagrelor in hemodialysis and healthy subjects. HD hemodialysis, SD standard deviation

The mean PRU was decreased following ticagrelor dosing, with the greatest change from baseline occurring approximately 2 h post-dose for all three treatments (Fig. 4). The PRU responses (both absolute values and changes from the baseline) were similar following ticagrelor pre- and post-hemodialysis, while there were much greater responses (lower absolute PRU values) observed in the healthy subjects compared with the hemodialysis subjects (Table 2). The PRU differences at baseline between hemodialysis subjects and healthy subjects contributed to the PRU difference observed at early time points after ticagrelor dosing. Additionally, the difference in effect of ticagrelor on PRU measured as the absolute PRU changes from their baselines is less pronounced which indicates that hemodialysis subjects did not have a greater PRU response when compared with healthy subjects.

### Safety

AEs were reported in seven individuals during the study treatment period, distributed across all three treatments (five hemodialysis subjects [three when ticagrelor was administered pre-hemodialysis and two when ticagrelor was administered post-hemodialysis] and two healthy subjects). There were no reports of bleeding or dyspnea in hemodialysis or healthy subjects who received ticagrelor. One subject who received ticagrelor post-hemodialysis reported potentially treatment-related AEs according to the investigator, comprising dizziness, bronchospasm, and nausea. This patient withdrew from the study before receiving the second ticagrelor dose. One other hemodialysis subject withdrew from the study because of a serious AE (thoracic vertebral fracture), which was not considered treatment related. There were no other serious AEs or deaths, and no clinically significant changes in laboratory parameters or vital signs were reported during the study.

## Discussion

This study investigated the PK, PD, and safety of ticagrelor in subjects with ESRD on hemodialysis and compared the results with those from healthy subjects with normal renal function. The effects of timing of hemodialysis on the PK of ticagrelor were also investigated as part of the study.

Overall, hemodialysis did not have a clinically significant impact on the exposure to ticagrelor or its active metabolite. The mean *C*_max_ and AUC_0-∞_ of ticagrelor were 61 and 51%, and 49 and 38% higher in pre- and post-hemodialysis subjects than in healthy subjects, respectively, while the mean *C*_max_ and AUC_0-∞_ of AR-C124910XX were 36 and 17%, and 14 and 13% higher in pre- and post-hemodialysis subjects, respectively, than that in healthy subjects. Notably, the mean *C*_max_ and AUC_0-∞_ of ticagrelor and AR-C124910XX were generally similar following a single 90-mg dose of ticagrelor 1 day after hemodialysis and 2 days prior to the next dialysis treatment, indicating that timing of hemodialysis has little effect on exposure to the drug or its metabolite. Moreover, the mean metabolite-to-parent *C*_max_ and AUC_0-∞_ ratios were comparable following all three treatments, suggesting that hemodialysis has little impact on the metabolic conversion of ticagrelor to AR-C124910XX.

The minimal impact of hemodialysis on ticagrelor PK observed in this study is consistent with the known elimination pathway of ticagrelor, which is predominantly via hepatic metabolism of ticagrelor to AR-C1249XX, followed by biliary secretion [5, 6]. Renal excretion plays only a minor role in the elimination of ticagrelor [5]. Moreover, as ticagrelor is highly protein bound (> 99.8%) [15], dialysis is unlikely to have an appreciable impact on its plasma concentration. The results of this study are also consistent with findings from subjects with severe renal impairment not on hemodialysis, which demonstrated exposure to ticagrelor and AR-C124910XX (20% lower and 17% higher, respectively, vs healthy subjects) to be largely unaffected by renal impairment [7].

IPA responses were also similar between hemodialysis and healthy subjects. Mean IPA time curves for all three treatments were almost indistinguishable, and final extent IPA was > 90% at 2 h post-dose in both hemodialysis and healthy subjects. Likewise, mean IPA_max_ and AUEC_0–48, IPA_ were similar across the three treatments. Median time to IPA_max_ was slightly longer when ticagrelor was administered just prior to hemodialysis versus 1 day after hemodialysis or in healthy subjects. However, time to IPA_max_ values in hemodialysis subjects displayed greater variability than those for healthy subjects, possibly reflecting the small sample size. Nevertheless, the *E*_max_ model parameters describing the PK/PD relationship between ticagrelor concentrations and IPA response were comparable across all three treatments. Of particular note, these findings show that hemodialysis subjects did not have greater responses to ticagrelor than healthy subjects, and that the timing of hemodialysis has little influence on the PD effect of ticagrelor on IPA.

For all three treatments, reductions in PRU were observed following ticagrelor dosing, with the greatest reductions observed at 2 h post-dose. The mean AUEC_0–48, PRU_ was of similar magnitude when ticagrelor was administered pre- or post-hemodialysis. However, there appeared to be a greater reduction in PRU in healthy subjects. This finding may be at least partially explained by baseline differences in PRU in hemodialysis versus healthy subjects that may have contributed to the PRU difference observed at early time points after ticagrelor dosing. Importantly, from a safety perspective in this special population, this study highlights that hemodialysis subjects showed no greater PRU response, compared with healthy subjects.

In this single-dose study, safety data were in line with previous studies of ticagrelor in healthy volunteers [16] and subjects with severe renal impairment not on dialysis [7]. AEs were reported in seven subjects across all treatment regimens—three subjects receiving ticagrelor pre-hemodialysis, two subjects receiving ticagrelor post-hemodialysis, and two healthy subjects. Similarly, vital signs and laboratory findings following ticagrelor administration were unremarkable in hemodialysis and healthy subjects, again consistent with previously reported findings in healthy [16] and renally impaired subjects [7].

In conclusion, the slightly higher exposure to ticagrelor and its active metabolite in hemodialysis subjects, compared with healthy subjects, is considered of minimal clinical relevance. Hemodialysis subjects had similar IPA response and no greater PRU response versus healthy subjects, and the safety profile was similar in both cohorts. Based on the PK, PD, and safety findings of this study, no ticagrelor dose adjustment is required for patients with ESRD on hemodialysis. These findings are consistent with the US Food and Drug Administration-approved label for ticagrelor, which suggests that even a twofold increase in exposure to ticagrelor does not warrant a dose adjustment [17]. Furthermore, the timing of hemodialysis has little effect on the PK and PD of ticagrelor.

## Electronic supplementary material

ESM 1 (DOCX 16 kb)
