# Supplementary material for: Pharmacokinetics and pharmacodynamics of ticagrelor in subjects on hemodialysis and subjects with normal renal function
Source: Eur J Clin Pharmacol. 2018 May 30;74(9):1141–8. doi: 10.1007/s00228-018-2484-7 (PMC6096709; doi:10.1007/s00228-018-2484-7)
Supplement: Supplementary file 1 — (DOCX 16 kb) [file 228_2018_2484_MOESM1_ESM.docx]

**Supplementary Materials**

**Table S1 Comparison of pharmacokinetic (PK) parameters (mean ratio [90% CI]) for ticagrelor and AR-C12490XX in hemodialysis versus healthy subjects**

| **PK parameter^a^** | **Pre-HD vs healthy subjects** | **Post-HD vs healthy subjects** |
| --- | --- | --- |
| **Ticagrelor** | | |
| C_max_ , ng/mL | 161.4 (122.5, 212.6) | 151.1 (112.0, 203.9) |
| AUC_0-∞_, ng·h/mL | 148.8 (115.1, 192.3) | 137.8 (105.7, 179.5) |
| **AR-C124910XX** | | |
| C_max_, ng/mL | 136.3 (95.4, 194.7) | 117.1 (84.5, 162.2) |
| AUC_0-∞_, ng·h/mL | 114.4 (91.2, 143.5) | 112.7 (88.6, 143.4) |

**AUC_0-∞_**, area under the concentration curve (AUC) from time zero to infinity; **CI,** confidence interval**; C_max_**, maximum observed plasma concentration; **HD**, hemodialysis; **HS**, healthy subjects

*****Values are geometric mean ratio, percent (90% confidence interval) for C_max_ and AUC_0-∞_
